# Molecular and Physiological Properties of Indigenous Strains of *Oenococcus oeni* Selected from Nero di Troia Wine (Apulia, Italy)

**DOI:** 10.3390/microorganisms10040795

**Published:** 2022-04-09

**Authors:** Maria Stella Cappello, Vittorio Falco, Rosita Curcio, Giovanni Mita, Giacomo Zapparoli

**Affiliations:** 1 CNR, Institute of Science of Food Production (ISPA), Prov.le Lecce-Monteroni, 73100 Lecce, Italy; vittorio.falco@ispa.cnr.it (V.F.); giovanni.mita@ispa.cnr.it (G.M.); 2 Department of Pharmacy, Health and Nutritional Sciences, University of Calabria, 87036 Rende, Italy; 3 Department of Biotechnology, University of Verona, Strada le Grazie 15, 37134 Verona, Italy; giacomo.zapparoli@univr.it

**Keywords:** *Oenococcus oeni*, Nero di Troia wine, amplified fragments length polymorphisms, genetic diversity, enzymatic activities, metabolic properties

## Abstract

The characterization of *Oenococcus oeni* strains isolated from Nero di Troia wine (Apulia, Italy) sampled in two distinct production areas was carried out. The two indigenous populations, consisting of 95 and 97 isolates, displayed high genetic diversity when analyzed by amplified fragments length polymorphisms (AFLP). Based on the UPGMA dendrogram obtained by AFLP analysis, the two populations displayed similar genotypes that grouped in the same clusters with a high level of similarity (>95%). One genotype was found in only one of the two areas. Representative strains of each cluster were analyzed for their enzymatic activities (esterase, β-glucosidase, and protease), assayed in whole cells, and tested for their metabolic properties (consumption of L-malic acid, citric acid, acetaldehyde, and arginine) and growth parameters. Significant differences among strains, including the reference strain ATCC BAA-1163, were observed for all of these properties. Principal component analysis evidenced phenotypic differences among strains, and well separated some of them belonging to different genotypes. Strains exhibiting the best performances in most of these traits could be further investigated in order to select possible candidates as malolactic starters for Nero di Troia wine. This study provided insights on the population structure of *O. oeni* of a local winemaking area useful to the understanding of the regional diversity of this bacterium, an issue not yet completely resolved

## 1. Introduction

*Oenococcus oeni* is the most important malolactic bacterium found in wine. A great number of studies documented its physiological and technological traits, which positively affect wine quality, as well as its genomic features contributing to its adaptation to the enological environment [1,2]. The important role of this bacterium in winemaking stimulated the individuation and selection of suitable strains to be used as culture starters in malolactic fermentation (MLF) in most wine areas of the world [3,4,5]. The investigations on indigenous *O. oeni* strains revealed that natural populations are characterized by high genetic polymorphism within a specific region or grape variety [1]. Although the terroir concept is debatable concerning wine microbiota, this regional intraspecific diversity contributes to the microbial wine’s terroir, and was exploited to valorize local products by winemaking communities [4,6,7,8].

Despite whole-genome studies contributed to the understanding of population structures of *O. oeni* in several winemaking areas by evidencing the presence of different genetic lineages [2], the regional polymorphism of this bacterium is still not completely resolved. Winery-specific genotypes of indigenous *O. oeni* can prevail over common genotypes within wineries of the same geographical region [9,10]. On the other hand, El Khrouy et al. [11] reported that strains isolated from four major winemaking regions of France are not genetically adapted to their origin areas, but to specific types of wine. Other investigations showed that the *O. oeni* population is completely adapted to winemaking regions [6,12]. Hence, further investigations on genetic and physiological polymorphisms of *O. oeni* associated with specific regions and types of wine are recommended.

Besides genetic/genomic diversity, evidence of variability on enological distinctive traits among indigenous strains contributed to select potential regional malolactic starters [4,7,8]. It is well documented that important technological properties such as resistance to various wine-limiting factors and metabolic parameters (e.g., malate and citrate consumption rate) are strain-dependent [13,14]. Moreover, strain diversity of enzymatic activities linked to aroma modification (e.g., esterase and β-glucosidase) was reported [15,16,17]. Hence, screening a pool of strains according to their main enological traits is a preliminary step required to obtain useful information on individual potential candidates for MLF culture starters.

In our previous investigations, indigenous strains of *O. oeni* isolated among the most common grape varieties of Apulia (Italy) such as Primitivo, Malvasia Nera, and Negroamaro were genetically and physiologically characterized [18,19,20]. High diversity within the sampling area was found, and no dominance of one or a few strains was observed. Indigenous *O. oeni* strains in combination with yeasts from Nero di Troia grape variety, which is commonly cultivated in Apulia region beside the above-mentioned varieties, were used as starters to produce local wines [21]. Their promising results encouraged further investigations to valorize the microbial terroir.

The aim of this study was the characterization of *O. oeni* strains isolated from wine obtained from the Nero di Troia grape variety. Two different areas of their production were sampled to investigate genetic and physiological polymorphic differences among strains. Strain typing was carried out by amplified fragments length polymorphisms (AFLP), a highly strain-discriminant tool. The enzymatic activities and metabolic properties of enological interest of such strains were assayed to evaluate their technological potential in order to select possible local malolactic bacterial starters.

## 2. Materials and Methods

### 2.1. Sampling and Strain Isolation

Two sampling areas of production of Nero di Troia wine, Castel del Monte (41°05′05.11″ N 16°16′15.37″ E) and San Severo (41°41′42.4″ N, 15°22′45.4″ E) (Apulia, Italy), were selected. The isolation of lactic acid bacteria was carried out from wine undergoing MLF produced in two wineries for each area during the 2019 vintage. Wine samples collected from wineries were transferred to the laboratory and spread in MRS (Millipore, Merck KGaA, Darmstadt, Germany), supplemented with 2% tomato juice (MRS-tj) at pH 4.8 and 2% agar, to which 0.1 mg mL^−1^ cycloheximide was added. Plates were incubated at 28 °C for 4–7 days in anaerobic conditions using the Anaerogen TM 2.5 L system (Thermo Fisher Scientific, Waltham, MA, USA). A total of 450 bacteria (210 from Castel del Monte and 240 from San Severo) were isolated by picking randomly the colonies grown on the plates. All isolates were maintained in MRS-tj broth and an aliquot with glycerol 25% (*v*/*v*) was stored at −80 °C.

### 2.2. Identification of Oenococcus oeni and Fluorescent AFLP Analysis

All 250 isolates were observed with an optical microscope and those with coccal-shaped cells were selected for total DNA extraction according to Cappello et al. [18]. These isolates were assayed for *O. oeni* identification by 16S rDNA sequencing using primers and PCR cycling parameters previously described [18]. Amplicons were analyzed by agarose gel electrophoresis, purified using the QIAquick spin Kit (Qiagen Science Inc., Germantown, MD, USA), and sequenced at the Eurofins Genomics service (Eurofins Genomics, Edersberg, Germany) using the same primers employed for amplification. Sequence similarity searches were carried out using a basic local alignment search (BLAST) in the EMBL/GenBank databases. AFLP fingerprinting of all *O. oeni* isolated from Nero di Troia wine and ATCC BAA-1163, used as a reference strain, and clustering by the unweighted pair group method with arithmetic mean (UPGMA), were performed as previously described [18].

### 2.3. Enzymatic Activity, Growth, and Metabolism Assays

Enzymatic activities (esterase, β-glucosidase and protease) were assayed spectrophotometrically on selected strains according to the AFLP dendrogram, and cells were prepared for each assay as described by Cappello et al. [19]. Esterase activity was assayed using 4-nitrophenyl-butyrate, 4-nitrophenyl-acetate, 4-nitrophenyl-octanoate, 4-nitrophenyl-decanoate, 4-nitrophenyl-myristate, and 4-nitrophenyl-dodecanoate (Merck KGaA). β-glucosidase activity was tested using three substrates (4-nitrophenyl-β-D-glucopyranoside, 4-nitrophenyl-β-D-mannopyranoside, and 4-nitrophenyl-N-acetyl-β-D-glucosaminide) (Merck KGaA), and protease activity was assayed using bovine serum albumin (BSA) and protein extracts from wine (WPE) obtained from a red table wine by extraction of the macromolecular fraction dialyzing against distilled water with membranes of 12 Kda for 48 h. Then, the retentate was lyophilized. Enzyme assays were carried out in triplicate.

Determination of specific growth rate and growth yield, as well as consumption of L-malic acid, citric acid, acetaldehyde, and arginine were carried out as previously reported [19]. Briefly, strains were grown in FT80 modified broth at pH 3.5 at 28 °C and periodically culture aliquots were sampled to determine the concentration of L-malic acid, citric acid, acetaldehyde, and arginine from supernatant obtained by centrifugation. L-malic acid, citric acid, and acetaldehyde were enzymatically measured by commercial kits (Megazyme, Neogen Europe Ltd., Auchincruive, Ayr, UK). The determination of arginine was performed according to Tonon and Lonvaud-Funel [22].

### 2.4. Statistical Treatment of Data

One-way analysis of variance (ANOVA) was used to compare mean values of enzymatic activities, growth parameters, as well as consumption of L-malic acid, citric acid, acetaldehyde, and arginine in order to evaluate significant differences in samples. A p-value less than 0.05 was considered as significant. Tukey’s multiple comparison test (Tukey, HSD) was applied to make pairwise comparisons. Principal component analysis (PCA) was performed using the statistical package XLSTAT 2017 (Addinsoft SARL, Paris, France) using the same values used for ANOVA. The analysis was carried out using data automatically scaled by the program (Pearson as PCA type).

## 3. Results

### 3.1. Identification and Typing of O. oeni Strains

**Figure 1:**
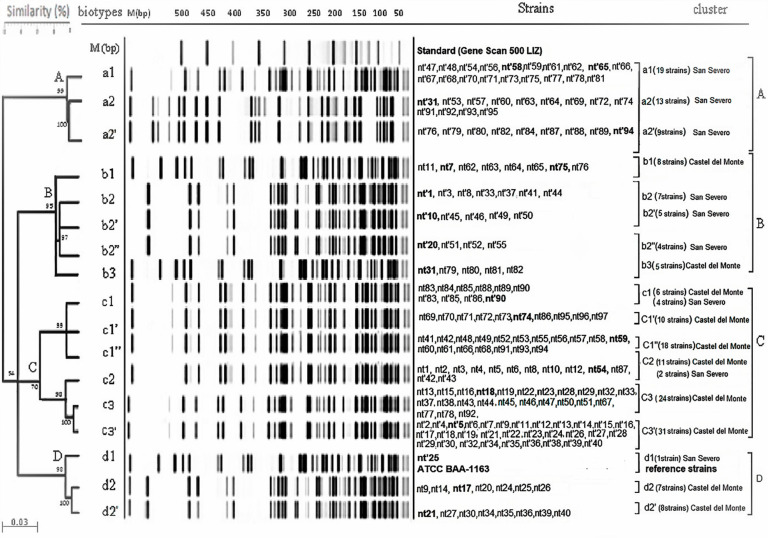
UPGMA dendrogram resulting from genotyping analysis performed by AFLP of 192 *O. oeni* isolates; “nt” and “nt’” indicate isolates from “Nero di Troia” wine from the Castel del Monte and San Severo area, respectively, while the number designates each strain. The strain ATCC BAA-1163 was used as a reference strain. In bold, the strains selected for the analysis of enzymatic activities, growth, and metabolic parameters.

A total of 192 indigenous *Oenococcus oeni* strains, isolated from two distinct areas of Nero di Troia winemaking region, were analyzed. In particular, 97 strains out of 210 lactic acid bacteria (LAB) and 95 out of 240 LAB were isolated from wine of the Castel del Monte and San Severo areas, respectively. These strains were firstly identified as *O. oeni* by 16S rDNA sequencing (data not shown); then, they were genetically characterized by AFLP analysis in comparison with the reference strain ATCC BAA-1163. Results were statistically analyzed by UPGMA cluster analysis using the Dice similarity coefficient, and the obtained dendrogram showed the genetic distances among each single genomic fingerprinting (Figure 1). Specifically, all isolates were split into four different main clusters named A, B, C, and D. 

A very high intraspecific homology (99–100%) was detected in cluster A, which comprised three biotypes all belonging to the San Severo area indicated as a1, a2, and a2’ and each one included 19, 13, and 9 isolates, respectively. However, cluster A evidenced 54% similarity to cluster C.

Cluster B comprised isolates from the two different areas; all 29 strains were split into five biotypes (b1, b2, b2’, b2’,’ and b3). Biotypes b1 (eight isolates) and b3 (five isolates) showed 95% similarity to each other, and they all belonged to the Castel del Monte area. Biotypes b2, b2’, and b2”, including seven, five, and four isolated, respectively, from the San Severo area, showed 97% similarity to each other. Instead, cluster B exhibited 70% similarity to cluster C.

Cluster C consisted of six biotypes called c1, c1’, c1”, c2, c3, and c3’. In the c1 biotype, the strains from the two different areas clustered; in detail, six strains were from Castel del Monte and four from the San Severo area. In the same way, in the c2 biotype, eleven strains from Castel del Monte and two from the San Severo area were included together. Furthermore, 99% homology was found between biotypes c1, c1’, and c1”; the similarity detected in the c2 biotype was also very high (98%) compared with that of c3 and c3’. 

Cluster D (16 isolates) displayed a very high AFLP similarity (98–100%) and consisted of three different subgroups designated as d1, d2, and d2’. All the strains were isolated from the Castel del Monte area, except for the nt’25 strain, which was derived from the San Severo area and was clustered in the d1 biotype together with the reference strain ATCC BAA-1163.

### 3.2. Enzymatic Activities 

Representative strains of each dendrogram cluster were chosen to analyze some enzymatic activities involved in the modification of wine aroma composition due to MLF. Esterase, β-glucosidase, and protease activities were assessed in whole cells of these strains, by using AWRI B429 as a reference strain. *Oenococcus oeni* strains displayed significant differences in each of these enzymatic activities (Table 1A,B).

Our results evidenced that the selected strains displayed significant esterase activity on four out of six substrates tested in the assay (Table 1A); indeed, no activity was detected on 4-N-myristate and 4-N-dodecanoate (data not shown). The highest activity was observed using 4-N-butyrate (average of all strains was 17.79 µmole/min/g dry weight), the lowest activity was detected using 4-N-decanoate (1.48 µmole/min/g dry weight as average). Almost all strains were pooled together with the reference strain, resulting from the statistical treatment of data, for activities assayed with 4-N-butyrate and 4-N-acetate. Conversely, most of the indigenous strains from Nero di Troia showed significant differences in esterase activity tested on 4-N-octanoate and 4-N-decanoate with respect to the reference strain.

All the tested strains showed β-glucosidase activity on 4-N-glucopyranoside (Table 1B), but not on 4-N-acetilpyranoside and 4-N-mannopyranoside (data not shown). This activity ranged from 0.68 (strain nt18) to 1.62 nmole/min/g dry weight (strain nt′1). Except for the strains nt’1 and nt’10, all strains displayed higher β-glucosidase activity than the reference strain.

Enzymatic differences among the selected strains were also observed for protease activity, assayed on two different substrates, BSA and WPE. Particularly, strains nt31, nt54, nt’25, and nt’31 displayed the highest activity, their values were similar to those found for the reference strain (215.16 and 216.10 nmole/min/g dry weight), while nt’7 and nt’5 strains exhibited the lowest protease activity on both substrates.

### 3.3. Growth and Metabolic Parameters

The selected strains significantly differed in their growth behavior evaluated by the determination of maximum growth rate and growth yield (Table 2A). Noteworthy, nt31, nt54, nt’20, nt’25, and nt’31 strains displayed optimal growth performance, with values of rate/yield growth very close to those of the reference strain.

Regarding metabolic parameters, strains displayed very different values of the consumption rate of L-malic acid and acetaldehyde, while differences in citric acid consumption rate were found to a lesser extent (Table 2B). Strains nt31, nt54, and nt’25 evidenced high consumption rates of L-malic acid, citric acid, and acetaldehyde. Anyway, although with significant differences, all the tested strains were able to metabolize the total amount of each metabolite supplemented in the growth medium, which was 5 g L^−1^, 0.5 g L^−1^, and 100 mg L^−1^ of L-malic acid, citric acid, and acetaldehyde, respectively (data not shown).

Regarding L-arginine metabolism, strains revealed significant differences, and most of them had values of consumed L-arginine lower than the reference strain (Table 2B).

### 3.4. Principal Component Analysis

**Figure 2:**
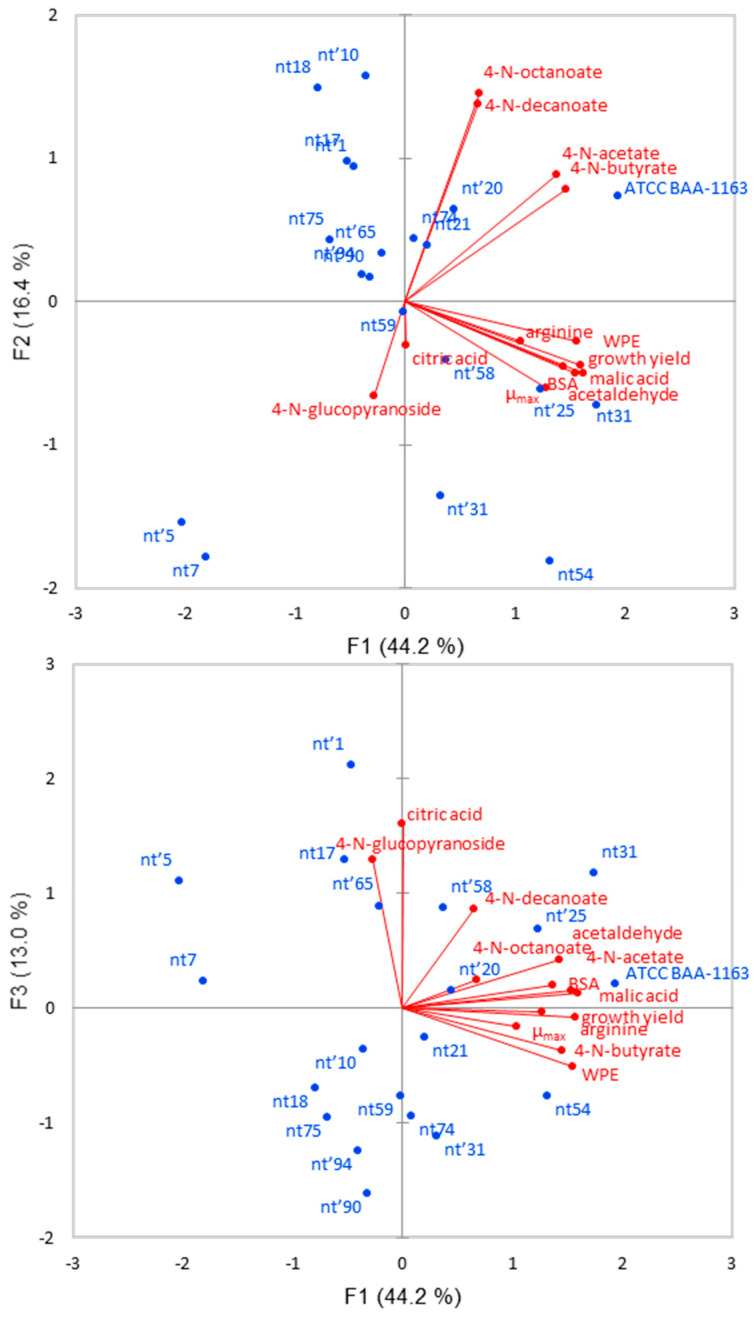
Principal component analysis bi-plot F1 and F2, and F1 and F3 obtained using physiological properties of 20 selected strains, according to UPGMA dendrogram and ATCC BAA-1163.

All 19 selected indigenous strains and the reference strain were analyzed by PCA using data on enzymatic activities and metabolic properties. A cumulative variability of 73.6% was explained by the first three factors (F1, F2, and F3). Seven parameters (esterase activity on 4-N-butyrate and 4-N-acetate, protease activity on BSA and WPE, growth yield, maximum L-malic acid, and acetaldehyde consumption rate) had a high loading score (>0.700) in F1, while two in F2 (esterase activity on 4-N-octanate and 4-N-decanoate). Some of these strains were well separated in both bi-plots (Figure 2).

In particular, nt31, nt54, and nt’25 were positioned on the right, due to high positive score values of F1, as well as the reference strain. On the contrary, strains nt’5 and nt’7 were well separated on the left due to negative F1 scores.

## 4. Discussion

Despite AFLP being widely recognized to have a very high discriminant power on strain typing [23], only a few studies on *O. oeni* populations, excluding our previous investigations [18,19,20], adopted this tool to analyze their diversity [24]. AFLP analysis of *O. oeni* strains isolated from Nero di Troia wine revealed that the two populations coming from different wine-producing areas are characterized by a high genetic diversity within each one. This observation is not a novelty considering our previous investigations carried out on indigenous *O. oeni* from three Apulia winemaking areas near that of this study [18,19,20]. Nevertheless, differently from these last investigations, the analysis of two distinct populations was carried out, and three out of four AFLP clusters contained isolates from both the Castel del Monte and San Severo areas. Hence, the lack of specific-genotypes for each of the two sampling areas confirms that there is no genetic adaptation of *O. oeni* strains to their local vineyard and winemaking conditions within each area. Rather, biotypes including isolates from both Castel del Monte and San Severo reveal the occurrence of predominant genotypes of the entire Nero di Troia winemaking region. The presence of the reference Australian strain ATCC BAA-1163, clustering with few indigenous strains (16 out of 192) at 54% similarity, suggests that *O. oeni* populations cannot be genetically distinguished according to their origin areas as previously described [11,13]. On the other side, the high frequency of certain genotypes, such as those clustering in A, B, and C in the present study, could indicate their better adaptation to specific types of wines. During MLF, the predominance of one genotype over another could be random, considering also that several of them display similar biochemical activities and growth parameters. 

Enzymatic activities linked to the release of aromatic compounds evidenced significant variability among the strains selected from the AFLP dendrogram. Quantitative variations for each assay were approximately similar to those observed among strains of the Malvasia Nera wine [19]. The strain-dependent capacity to release aroma compounds in wine was widely documented [14,15,24]. Nevertheless, Nero di Troia strains did not greatly differ among them about enzymatic activities, at least in a statistically significant way. Wide differences in β-glucosidase and esterase activity among *O. oeni* strains of different origins were reported in previous studies [15,16,17,25]. These differences could be related to the origin of the strains. A greater divergence in metabolic activities could be expected among strains of very different origins, such as those isolated from different countries than those coming from a specific area, such as the Nero di Troia winemaking area. Nevertheless, the variability on enzymatic activities among the strains of this study, which contributes to enhance wine aroma complexity, could be higher using other substrates and assay conditions. Diez-Ozaeta et al. [5] reported great variation in enzymatic activities among strains from the Rioja Alavesa winemaking region depending on pH and ethanol concentration combinations. Conversely, the substantial differences observed in the consumption rates of L-malic acid and acetaldehyde among some strains of Nero di Troia are congruent with previous data [5,8,26]. From a technological point of view, strain variability concerning these performances was an important source in the selection of new starters to enhance aroma complexity in the Nero di Troia wine region [21,26]. If high L-malic degradation ability by starter strains is recommended to reduce the malolactic fermentation time, the capacity to degrade SO_2_-bound acetaldehyde, which affects the wine color, could be an undesirable property [27]. However, since Nero di Troia wine is characterized by an intense color, this character could be less relevant for the strain selection. Regarding citrate metabolism, the different ability of strains to degrade it could have significant effects on the sensory quality of wine [28]. The increase in acetic acid, diacetyl, and other acetoinic compounds could be expected from strains with a high citrate metabolism rate, such as nt’1, nt’5, and nt17. Concerning arginine metabolism, possible implications on microbiological stability depending on strain activity cannot be excluded, since arginine is an energy source for spoilage microorganisms [29]. As supported by PCA, strains nt’58, nt31, nt54, and nt’25 seem to be representative of each of the four AFLP clusters, and they have the best potentialities to be further investigated as potential malolactic starters.

## 5. Conclusions

This preliminary study on the *O. oeni* population of the Nero di Troia winemaking region evidenced the high diversity and specificity of indigenous strains through strain typing by a discriminant tool such as AFLP and by biochemical characterization. Since the two distinct sampling areas shared the same genotypes, a strain adaptation to specific vineyard and winery conditions was excluded. Rather, the presence of pools of strains characterized by different biochemical performances provided interesting information on the *O. oeni* population structure of the Nero di Troia winemaking area. The approach here described allowed the individuation of potential new starters within this strain pool. Further investigations are needed to evaluate these strains for other important biochemical activities related to wine spoilage such as acetic acid production, or the formation of undesirable compounds for consumers such as biogenic amines and ethyl carbamate. Finally, assays on the performance of these cultures at winery conditions should be conducted, with a particular focus on their ability to maintain the typicality of wine through the analysis of the main aroma compounds that contribute to the specific sensory quality of Nero di Troia wine.

## Figures and Tables

**Table 1:** (**A**). Esterase (µmole/min/g dry weight) activity measured in whole cells of 19 *O. oeni* strains isolated from Nero di Troia wine (nt, Castel del Monte and nt’, San Severo) selected according to AFLP analysis. ATCC BAA-1163 was used as a reference strain. Results were expressed as means ± SD. (**B**). Glucosidase (µmole/min/g dry weight) and protease activity (nmole/min/g dry weight) measured in whole cells of 19 *O. oeni* strains isolated from Nero di Troia wine (nt, Castel del Monte and nt’, San Severo), selected according to AFLP analysis. ATCC BAA-1163 was used as a reference strain. Results are expressed as means ± SD.

(**A**)				
**Strain**	**Esterase**			
	**4-N-Butyrate**	**4-N-Acetate**	**4-N-Octanoate**	**4-N-Decanoate**
ATCC BAA-1163	19.40 ± 0.28 a	13.83 ± 0.81 a	6.98 ± 0.44 a	1.77 ± 0.079 ab
nt7	10.92 ± 0.43 b	8.66 ± 0.75 de	2.87 ± 0.10 f	1.00 ± 0.02 no
nt17	18.26 ± 1.00 a	13.36 ± 0.54 ab	5.04 ± 0.28 bcd	1.60 ± 0.04 bcd
nt18	18.59 ± 1.21 a	12.63 ± 0.90 ab	5.24 ± 0.20 bcd	1.44 ± 0.05 defg
nt21	18.72 ± 1.23 a	12.46 ± 0.85 abc	5.34 ± 0.25 bc	1.46 ± 0.06 cdef
nt31	20.11 ± 0.72 a	13.55 ± 0.79 ab	5.07 ± 0.22 bcd	1.60 ± 0.05 mno
nt54	19.69 ± 0.76 a	13.09 ± 0.17 ab	2.94 ± 0.45 f	1.00 ± 0.07 o
nt59	18.13 ± 0.65 a	11.83 ± 0.77 abc	4.51 ± 0.17 de	1.31 ± 0.04 fghil
nt74	18.28 ± 1.16 a	12.51 ± 0.84 abc	5.23 ± 0.20 bcd	1.39 ± 0.05 efghi
nt75	18.13 ± 1.07 a	11.50 ± 0.59 bc	4.99 ± 0.20 cd	1.23 ± 0.04 hilm
nt’1	17.46 ± 1.10 a	13.50 ± 0.58ab	5.08 ± 0.27 bcd	1.77 ± 0.06 ab
nt’5	11.65 ± 0.51 b	8.03 ± 0.78 e	3.00 ± 0.11 f	1.12 ± 0.04 lmno
nt’10	18.65 ± 1.16 a	12.51 ± 0.89abc	4.94 ± 0.21 cd	1.87 ± 0.06 a
nt’’20	18.51 ± 1.26 a	12.62 ± 0.89 ab	5.79 ± 0.27 b	1.65 ± 0.08 bc
nt’25	20.04 ± 0.80 a	12.41 ± 0.77 abc	5.12 ± 0.22 bcd	1.50 ± 0.08 cdef
nt’31	18.51 ± 0.71 a	12.59 ± 0.16 ab	2.01 ± 0.40 g	1.10 ± 0.06 bc
nt’58	17.78 ± 0.72 a	12.47 ± 0.84 abc	4.00 ± 0.13 e	1.51 ± 0.06 cde
nt’65	17.94 ± 1.10 a	12.54 ± 0.84 abc	5.38 ± 0.22 bc	1.42 ± 0.12 defgh
nt’90	17.77 ± 1.11 a	11.98 ± 0.60 abc	4.82 ± 0.18 cd	1.20 ± 0.04 ilmn
nt’94	18.80 ± 1.13 a	10.32 ± 0.62 cd	4.90 ± 0.20 cd	1.26 ± 0.09 ghilm
(**B**)				
**Strain**	**β-Glucosidase**		**Protease**	
	**4-N-glucopiranos**		**BSA**	**WPE**
ATCC BAA-1163	0.76 ± 0.04 ab		215.16 ± 6.21 a	216.10 ± 1.47 a
nt7	1.45 ± 0.04 ilm		98.49 ± 5.12 f	99.37 ± 4.84 f
nt17	1.46 ± 0.05 lm		142.38 ± 3.8 bcd	163.72 ± 6.37 d
nt18	0.68 ± 0.06 a		107.59 ± 2.12 f	122.82 ± 4.56 e
nt21	1.18 ± 0.04 fgh		139.89 ± 2.24 cde	190.99 ± 7.40 bc
nt31	1.58 ± 0.07 mn		214.62 ± 5.82 a	207.56 ± 5.77 ab
nt54	1.02 ± 0.04 cdef		211.70 ± 5.30 a	217.78 ± 2.88 a
nt59	1.11 ± 0.04 defg		130.85 ± 2.08 de	174.46 ± 6.77 cd
nt74	1.17 ± 0.04 efgh		139.46 ± 2.22 cde	189.22 ± 6.60 c
nt75	1.01 ± 0.04 cde		133.74 ± 2.08 cde	177.59 ± 6.39 cd
Nt’1	1.62 ± 0.06 n		153.91 ± 4.30 b	101.22 ± 5.63 f
nt’5	1.57 ± 0.04 mn		99.46 ± 5.69 f	100.90 ± 4.95 f
nt’10	0.88 ± 0.06 bc		105.85 ± 2.028 f	121.95 ± 4.33 e
nt’20	1.29 ± 0.05 hi		144.83 ± 2.38 bc	189.10 ± 6.91 c
nt’25	1.55 ± 0.09 mn		211.28 ± 5.53 a	221.99 ± 5.24 a
nt’31	1.10 ± 0.04 defg		206.60 ± 5.14 a	212.25 ± 2.04 a
nt’58	1.33 ± 0.06 hil		129.95 ± 1.91 e	167.93 ± 5.43 d
nt’65	1.59 ± 0.03 mn		140.46 + 2.40 cde	178.13 ± 6.18 cd
nt’90	1.26 ± 0.06 gh		136.00 ±1.97 cde	166.78 ± 6.11 d
nt’94	1.00 ± 0.04 cd		135.63 ± 2.28 cde	175.85 ± 6.22 cd

Letter within each column means that values are significantly different for *p* < 0.05.

**Table 2:** (**A**). Growth parameters (maximum growth rate, µ_max_ h^−1^ and growth yield, g dry weight L^−1^) of 19 *O. oeni* strains isolated from Nero di Troia wine (nt, Castel del Monte and nt’, San Severo), selected according to AFLP analysis. ATCC BAA-1163 was used as a reference strain. Results are expressed as means ± SD. (**B**). Metabolic parameters (maximum consumption rates of L-malic acid, citric acid, and acethaldehyde, mgL^−1^ h^−1^, and percentage of arginine consumed) of 19 *O. oeni* strains isolated from Nero di Troia wine (nt, Castel del Monte and nt’, San Severo), selected according to AFLP analysis. ATCC BAA-1163 was used as a reference strain. Results are expressed as means ± SD.

(**A**)				
**Strain**	**µ_max_ (h^−1^)**		**Growth Yield**	
ATCC BAA-1163	0.005 ± 0.0007 abc		0.150 ± 0.004 a	
nt 7	0.004 ± 0.0007 ab		0.095 ± 0.004 e	
nt17	0.003 ± 0.0007 a		0.096 ± 0.004 e	
nt18	0.003 ± 0.0007 a		0.098+ 0.005 e	
nt21	0.005 ± 0.001 abc		0.120 ± 0.008 cd	
nt31	0.007 ± 0.001 c		0.150 ± 0.005 a	
nt54	0.006 ± 0.0007 bc		0.149 ± 0.001a	
nt59	0.004 ± 0.0007 ab		0.122 ± 0.009 cd	
nt74	0.004 ± 0.0007 ab		0.116 ± 0.010 cd	
nt75	0.005 ± 0.0007 abc		0.093 ± 0.005 e	
nt’1	0.003 ± 0.0007 a		0.106 ± 0.004 de	
nt’5	0.003 ± 0.0007 a		0.097 ± 0.005 e	
nt’10	0.004 ± 0.001 ab		0.125 ± 0.004 bc	
nt’20	0.004 ± 0.0007 ab		0.148 ± 0.004 a	
nt’25	0.006 ± 0.0007 bc		0.150 ± 0.005 a	
nt’31	0.004 ± 0.0007 ab		0.147 ± 0.001 a	
nt’58	0.003 ± 0.0007 a		0.141 ± 0.004 ab	
nt’65	0.003 ± 0.0007 a		0.097 ± 0.006 e	
nt’90	0.003 ± 0.0008 a		0.116 ± 0.011 cd	
nt’94	0.003 ± 0.001 a		0.110 ± 0.004 cde	
(**B**)				
**Strain**	**L-Malic Acid**	**Citric Acid**	**Acetaldehyde**	**ArginineConsumed**
ATCC BAA-1163	171.0 ± 4.1 a	4.4 ± 0.2 cdefg	2.6 ± 0.1 a	48 ± 2 a
nt 7	50.9 ± 4.2 g	4.2 ± 0.3 bcdefg	0.8 ± 0.07 efg	26 ± 4 fghi
nt17	25.7 ± 1.6 h	5.0 ± 0.3 fg	0.5 ± 0.1 gh	24 ± 3 ghi
nt18	23.8 ± 1.8 h	4.1 ± 0.4 bcdef	0.6 ± 0.07 fgh	23 ± 4 ghi
nt21	84.9 ± 3.8 f	4.0 ± 0.2 bcde	0.9 ± 0.07 def	31 ± 3 defg
nt31	169.6 ± 4.4 a	4.6 ± 0.3 defg	2.1 ± 0.1 b	44 ± 3 ab
nt54	165.5 ± 4.1 a	4.3 ± 0.2 cdefg	2.0 ± 0.1 bc	41 ± 4 abc
nt59	89.7 ± 2.9 ef	3.7 ± 0.3 bc	1.1 ± 0.07 de	38 ± 3 bcd
nt74	84.8 ± 3.7 f	3.3 ± 0.3 ab	0.9 ± 0.1 def	36 ± 4 bcde
nt75	24.5 ± 1.6 h	3.9 ± 0.4 bcd	0.1 ± 0.03 i	20 ± 1 i
nt’1	105.3 ± 2.4 d	5.0 ± 0.3 fg	0.5 ± 0.07 gh	21 ± 4 hi
nt’5	24.9 ± 1.7 h	5.0 ± 0.3 g	0.5 ± 0.07 gh	24 ± 4 ghi
nt’10	24.8 ± 1.8 h	3.7 ± 0.2 bc	0.7 ± 0.1 fgh	30 ± 3 efgh
nt’20	152.0 ± 5.0 b	4.0 ± 0.2 bcde	0.5 ± 0.2 gh	28 ± 2 efghi
nt’25	173.4 ± 4.4 a	4.3 + 0.2 cdefg	1.6 ± 0.07 c	22 ± 1 hi
nt’31	90.1 ± 3.7 ef	4.0 ± 0.3 bc	0.5 ± 0.1 gh	33 ± 2 cdef
nt’58	126.5 ± 3.6 c	4.8 ± 0.4 efg	1.8 ± 0.1 bc	48 ± 1 a
nt’65	27.4 ± 3.7 h	4.6 ± 0.3 defg	1.2 ± 0.2 d	47 ± 1 a
nt’90	89.3 ± 3.0 ef	2.6 ± 0.1 a	0.6 ± 0.07 fgh	37 ± 3 bcde
nt’94	99.6 ± 4.1 de	3.6 ± 0.3 bc	0.4 ± 0.1 hi	35 ± 3 cde

Letter within each column means that values are significantly different for *p* < 0.05.

## Data Availability

Not applicable.
